# A BIL Population Derived from *G*. *hirsutum* and *G*. *barbadense* Provides a Resource for Cotton Genetics and Breeding

**DOI:** 10.1371/journal.pone.0141064

**Published:** 2015-10-30

**Authors:** Xinhui Nie, Jianli Tu, Bin Wang, Xiaofeng Zhou, Zhongxu Lin

**Affiliations:** 1 National Key Laboratory of Crop Genetic Improvement, Huazhong Agricultural University, Wuhan, 430070, Hubei, China; 2 Cotton Institute, Xinjiang Academy of Agriculture and Reclamation Science, Shihezi, 832000, Xinjiang, China; USDA-ARS-SRRC, UNITED STATES

## Abstract

To provide a resource for cotton genetics and breeding, an interspecific hybridization between *Gossypium hirsutum* cv. Emian22 and *G*. *barbadense* acc. 3–79 was made. A population of 54 BILs (backcross inbred lines, BC_1_F_8_) was developed with the aim of transferring *G*. *barbadense* genes into *G*. *hirsutum* in order to genetically analyze these genes’ function in a *G*. *hirsutum* background and create new germplasms for breeding. Preliminary investigation of the morphological traits showed that the BILs had diverse variations in plant architecture, seed size, and fuzz color; the related traits of yield and fiber quality evaluated in 4 environments also showed abundant phenotypic variation. In order to explore the molecular diversity of the BIL population, 446 SSR markers selected at an average genetic distance of 10 cM from our interspecific linkage map were used to genotype the BIL population. A total of 393 polymorphic loci accounting for 84.4% MAF (major allele frequency) > 0.05 and 922 allele loci were detected, and the Shannon diversity index (I) was 0.417 per locus. The average introgression segment length was 16.24 cM, and an average of 29.53 segments were introgressed in each BIL line with an average background recovery of 79.8%. QTL mapping revealed 58 QTL associated with fiber quality and yield traits, and 47 favored alleles derived from the donor parent were discovered. This study demonstrated that the interspecific BIL population was enriched with much phenotypic and molecular variation which could be a resource for cotton genetics and breeding.

## Introduction

Cotton is one of the most important crops in the world, providing natural fiber for the textile spin industry. Therefore, the development of cotton production is of great importance. In particular, germplasm innovation plays a decisive role in improving new cotton varieties. Since the 1950s, many cotton germplasms have been developed in China. *G*. *hirsutum* accounts for 95% of the cotton planting area [1]. Although the planting of *G*. *barbadense* accessions has been limited, they contain elite genes related to fiber quality traits, and cotton breeders are attempting to introduce them into *G*. *hirsutum* to improve the fiber quality traits of *G*. *hirsutum* while maintaining its high yield performance.

Before applying these preferred genes, it is necessary to genetically map them. Some researches have been reported using interspecific high generation backcross populations to detect main-effect QTL, such as in rice [2,3], barley [4] and wheat [5]. In cotton, the genetic basis of high-generation lines between *G*. *hirsutum* and *G*. *barbadense* could be used to detect main-effect QTL, and also to breed good varieties with potential application in production. Many researchers have used the populations derived from *G*. *hirsutum* and *G*. *barbadense* to detect QTL related to agronomic traits [6], fiber quality traits [7–9], and verticillium wilt resistance [10,11].

BILs (backcross inbred lines) are developed through one backcross and several generations’ selfing, during which no phenotype or marker-assisted selections are conducted. Generally, BILs contain many variations and are important resources for both genetic research and breeding projects. BILs can be used to preliminarily map QTL, and fine mapping can subsequently be conducted by developing advanced segregation populations. In order to apply elite alleles of *G*. *barbadense* to genetically improve *G*. *hirsutum* (especially its fiber quality), a BIL population was developed. In this study, *G*. *hirsutum* cv. Emian22 as a recurrent parent was crossed with *G*. *barbadense* acc. 3–79 as a donor and backcrossed for one generation; it was then continuously self-crossed to create the BIL population. In order to evaluate the potential applications for genetics and breeding, this BIL population was genotyped by genome-wide SSR markers that were selected from the BC_1_ interspecific linkage map constructed based on the same parents and phenotyped under multi-environments. This study aimed to (1) explore the molecular diversity of the BILs; (2) evaluate the variations in agronomic and fiber quality traits; (3) identify molecular markers associated with phenotypic variations; and (4) provide materials for future cotton genetics and breeding.

## Materials and Methods

### Plant materials and field experiments

The F_1_ generation was developed by crossing Emian22 as the female parent with 3–79 as the male parent, during the summer of 2006 in the fields of Huazhong Agricultural University in Wuhan, Hubei province. It was then backcrossed with Emian22 as the male parent to produce the BC_1_ population during the winter of 2006 in Sanya, Hainan province. Finally, the BC_1_ was planted and self-crossed during the summer of 2007 in Wuhan, Hubei province. In subsequent years, the population was self-pollinated in Wuhan, Hubei province, and in Sanya, Hainan province. Finally, 54 BC_1_F_8_ BILs were obtained. The BILs field experiment was carried out with two replicates in Jingzhou, Hubei province (in 2011 and 2012), Huangzhou, Hubei province (in 2011), and Shihezi, Xinjiang (in 2012). Different regions had different cultivation management patterns: in both Jingzhou and Huangzhou, the row spacing was 100 cm, with 40 cm between individuals and 10 individuals per 5 meters in one row, seedlings were transplanted and no irrigation was used. In Shihezi, the row spacing was (40+50+46) cm, with 9.5 cm between individuals and 100 individuals per 5 meters in one row; seeds were planted by sowing in a hole and drip irrigation was used. These fields are only used for research purposes, and the field studies did not involve endangered or protected species. Jingzhou in 2011, Huangzhou in 2011, Jingzhou in 2012 and Shihezi in 2012 were recorded as E1, E2, E3 and E4, respectively.

Plant height (PH), yield and fiber quality traits were measured in mid-September of 2011 and 2012. A total of 100 bolls from each line were collected for determining lint percentage (LP), seed cotton weight per boll (BW), boll number per plant (BN) and seed index (SI). To measure fiber quality traits, 10–15 g fibers was collected from middle fruit branches and sent to the Cotton Fiber Test Center at the Ministry of Agriculture, Anyang, Henan province, China. The tests were conducted using the HVI900 fiber quality detector at a temperature of 20°C with 65% relative humidity. Fiber quality traits included fiber upper half mean length (FUHML), fiber uniformity (FU), fiber strength (FS), fiber elongation (FE), and micronaire value (MV).

### SSR marker analysis

SSR markers were selected at an average of 10 cM from the BC_1_ interspecific linkage map of *G*. *hirsutum* cv. Emian22 × *G*. *barbadense* acc. 3–79 constructed in our laboratory [12]. A total of 446 markers, which were equally distributed on the 26 chromosomes, were selected to detect the BILs. Polymerase chain reaction (PCR), electrophoresis and silver staining were performed according to the protocols described by Wang et al. [13].

### Phenotypic statistical analysis

SPSS 18.0 software (http://www-01.ibm.com/software/analytics/spss/) was used to calculate basic statistics, including mean, maximum, minimum, standard value, skewness and kurtosis value. Moreover, the variable coefficient was analyzed using DPS software. Finally, the boxplot figures of 10 traits in 4 environments were analyzed using GraphPad Prism version 5.00 for Windows, GraphPad Software (San Diego, California, USA, www.graphpad.com).

The NTSYS-pc 2.10e [14] statistical package was used for analysis of phenotypic clustering, Euclid was used as a calculation function of Euclidean distance, and UPGMA was used as the clustering method.

### Genetic diversity analysis

The Shannon index (I), number of valid alleles (Ne), observed heterozygosity values (Ho) and expected heterozygosity (He) were estimated using the AMOVA function of GENA- LEX 6.2 software [15].

The genetic similarity matrices were determined by using the NTSYS-pc 2.10e statistical package based on Jaccard’s algorithms and the UPGMA clustering method [14].

### Genotype analysis

Graphical Genotype32 software was used to analyze introgression fragments from 3–79 with Emian22 as the background, based on the SSR markers’ location on the interspecific linkage map of *G*. *hirsutum* × *G*. *barbadense*. The same genotypes as Emian22 and 3–79 were recorded as A and B, respectively; the heterozygous genotype was recorded as H, the emergence of a new type band was recorded as N, and missing data were recorded as M. The length of introgression fragments was calculated, and a pictorial diagram of genotypes was drawn. The length of introgression fragments (cM) = the length of homozygous introgression fragments +1/2 hybrid import fragment length.

Both the statistics for the number of introgression fragments and the calculation of recovery rate of background were performed using MapDisto Genetics Software: CSSL Finder (http://mapdisto.free.fr/CSSLFinder/).

### Segregation distortion and hot introgression regions

In order to investigate the segregation distortion markers and hot introgression regions, a ⅹ^2^ analysis was performed in BILs to test whether the number of lines with A, B and H genotypes fit with the expected values of the BC_1_F_8_ generation. Hot introgression regions were defined as those chromosome regions containing at least three adjacent markers that were significantly segregation distorted (P<0.05) [16].

### Association analysis with yield and fiber quality traits

Marker loci with MAF<0.05 were eliminated in order to reduce false positive associations; single-marker variance analysis based on genotype and phenotype was performed on the remaining 330 polymorphic marker loci using SPSS 18.0 software. The markers that were significantly associated with phenotypic traits were selected, and the P value was corrected by FDR [17], where P≤0.01 was considered a significant association. Function annotation and expression of association loci were performed using BLAST tools to search the TM-1 genome sequence reported by Zhang et al. [18].

The distribution mapping of associated markers on the linkage map of *G*. *hirsutum* × *G*. *barbadense* cotton [12] was drawn using MapChart2.1 software [19].

### Analysis of allelic variation in associated loci

After association analysis, the bands of recurrent parent Emian22 and other non-parents were recorded as null alleles; phenotypic effects [10, 20] of allelic variance from 3–79 were then calculated using the following formulas:

α_i_ = Σx_ij_ / n_i_ -ΣN_k_/n_k_, where α_i_ denotes the phenotypic effects of the i allelic variance from 3–79, x_ij_ denotes the phenotypic value of the j material carried with the i allelic variance, and n_i_ denotes the number of materials of the i allelic variance. N_k_ refers to the number of materials of the null allelic variance. If α_i_ equated to positive values, the phenotypic effects of allelic variance from 3–79 signified an increasingly effective allele; otherwise, it indicated decreasing effectiveness of the allele.

## Results

### Phenotypic variation

**Fig 1:**
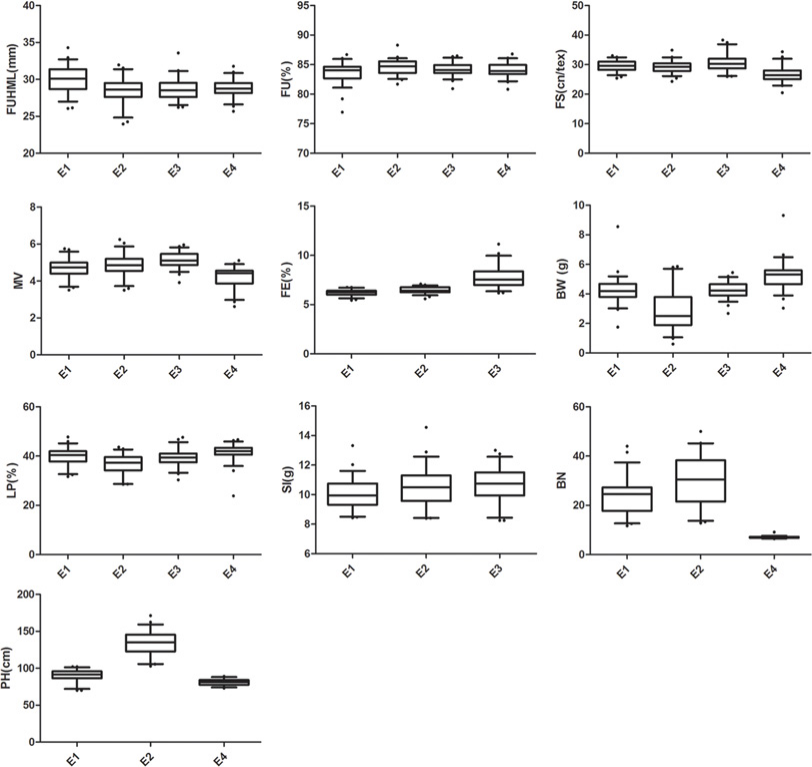
The box plot figures of ten traits in four environments.

Initially, nearly 200 BC_1_ plants were used to develop the BIL population; however, after several generations of selfing, only 54 BILs were obtained due to unavailable seeds, late maturation, weak vigor, etc. The BILs were used to evaluate the phenotypic variations of 10 traits, including 5 fiber quality traits, 4 yield-related traits and plant height. These traits were analyzed using SPSS version 18.0 and DPS version 7.05 (Fig 1 and S1 Table). Among the fiber quality traits in 4 environments, 16 group data showed normal distribution, except FUHML (E3), FU (E1) and FS (E4), while 9 yield group data showed normal distribution, except LP (E4), BW (E1; E4), BN (E4) and SI (E2). Plant height was normally distributed. Meanwhile, the standard deviation and variation coefficient showed that phenotypic variation was abundant, with variation coefficients ranging from 1.34% (FU in E3 and E4) to 47.24% (BW in E2). The variation coefficients were different for different traits: the minimum value of variation coefficients was FU (1.520) and the maximum value was BW (24.428) on average. CV% of BILs indicated that the agronomic traits of BILs were very different for each BIL (Fig 1).

Although we did not investigate other plant traits under multiple environments, the preliminary survey of plant architecture traits at Huazhong Agricultural University, Wuhan Hubei province in 2011 showed that these BILs were highly varied in plant size, leaf shape, boll shape and size and fuzz color (S1 Fig), etc (S2 Table).

### Phenotypic clustering

Many of the 36 phenotypic group datasets from the 54 BILs in four environments were used to study genetic diversity of phenotypes through the calculation of Euclidean distance as well as through clustering analysis (S2 Fig). The mean value of Euclidean distance between every two lines in the 54 BILs was 8.13; the maximum value of Euclidean distance between BS10 and BS41 was 13.30, and the minimum between BS34 and BS55 was 2.84. This indicated that the phenotype of BS34 was nearest to BS55 instead, and the maximum phenotype difference existed between BS10 and BS41 across different environments.

### SSR-based molecular diversity

Based on our previously published genetic linkage map information derived from *G*. *hirsutum* × *G*. *barbadense* [12], SSR markers at an average of 10 cM were selected, resulting in 446 SSR markers. Among them, 391 SSR markers were polymorphic in the BILs with 36 SSR markers having only one type of fragment derived from Emian22. The un-introgression gaps, accounting for 8.4% of the successfully amplified SSR markers, were distributed on 18 chromosomes, in which the maximum number of missing markers was 4 introgression loci for both chromosomes 15 and 22, but there was no obvious tendency in terms of concentration of the missing markers. A total of 922 alleles were obtained from the 391 SSR markers, of which 10 loci belonged to rare alleles (Minor allele frequency, MAF<0.01), 51 loci with minor allele frequency (MAF) were less than 0.05, and the rest of the 330 loci (84.4%) with MAF were greater than 0.05. The average effective number of alleles (Ne) was 1.37, the average observed heterozygosis (Ho) was 0.05, the Shannon index (I) was 0.42, and the overall expected heterozygosity (He) was 0.24.

NTSYS software was used to analyze the genotypic data of 922 alleles and to obtain both the genetic similarity coefficient matrix and the UPGMA clustering figure. The results showed that no subpopulations were determined by the clustering method among the BILs because they shared many loci and had a high genetic background recovery rate from Emian22 (S3 Fig). However, some valuable information was obtained: the average value of the genetic similarity coefficient between two BILs was 0.64; the maximum genetic similarity coefficient (coefficient = 0.87) was between BS14 and BS54, and the minimum value (coefficient = 0.48) was between BS20 and BS30. BS10 had the maximum genetic similarity coefficient (coefficient = 0.88) with the receptor parent Emian22, while BS30 had the minimum genetic similarity coefficient (coefficient = 0.60) with the receptor parent Emian22.

### Introgression analysis

**Fig 2:**
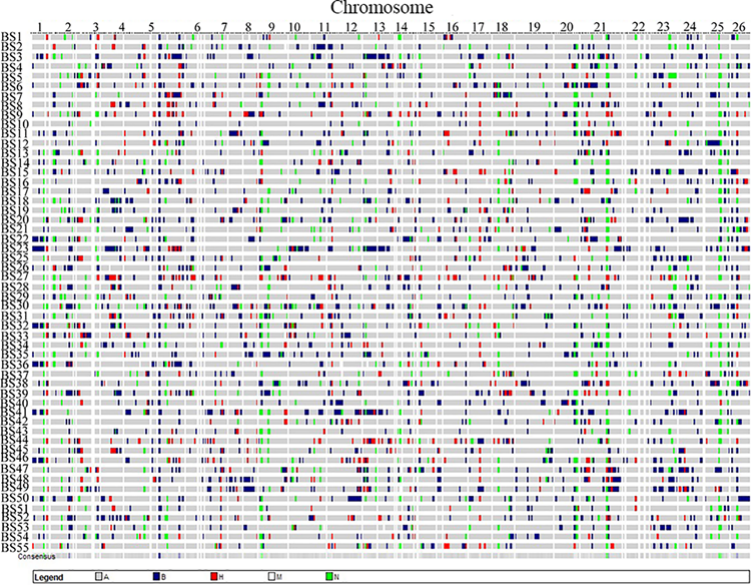
Graphic genotypes of the 54 BILs.

Based on our interspecific linkage map [12], there were an average of 17.15 introgression loci on each chromosome, with a range between 11 and 26 (Fig 2). The average background recovery rate was 79.8% for every line, distributing between 69.51% and 89.25%. The number of total introgression fragments in the 54 BILs was 1595, with an average of 29.53 per line, ranging between 15 and 52 (S3 Table). The total introgression length was 25904.2 cM, accounting for 5.86 times the total length of the reference map; the average introgression length was 479.71 cM per line, accounting for 10.8% of the genome length, ranging from 278.15 cM on Chr.21 to 2193.10 cM on Chr. 22. The average number and length of introgression fragments per chromosome was 61.35 (between 24 and 125) and 16.22 cM (between 10.58 cM and 25.72 cM), respectively. Segregation distortion analysis was performed using the chi-square test (P<0.05), and the chromosome regions with three adjacent segregation distortion loci were considered hot introgression regions. Among the 391 SSR markers with polymorphism, only 60 SSR markers showed no segregation distortion and 331 showed significant segregation distortion. A total of 39 hot segregation distortion regions were distributed on 18 chromosomes. A total of 187 loci were partial to the 3–79 genotype, 35 loci were significantly partial to the heterozygous genotype, and 108 loci were partial to both the 3–79 and heterozygous genotypes (S4 Table).

### QTL mapping

**Fig 3:**
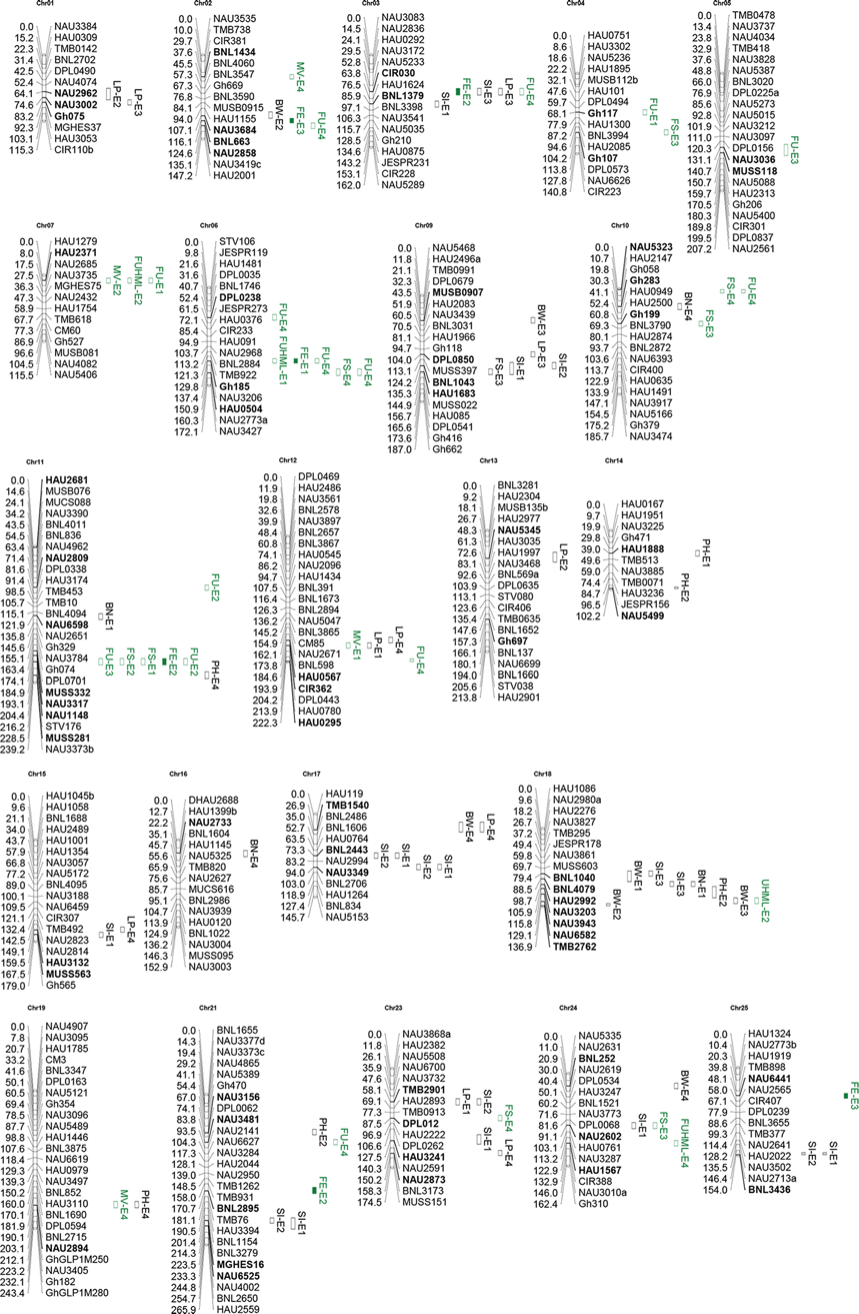
The localization of QTL in the linkage map.

The ANOVA analysis was conducted on phenotypic traits over the two years of the study using SPSS 18.0, and the polymorphic loci with MAF<0.05 were omitted. A total of 58 marker loci significantly associated with PH, yield and fiber quality traits were detected in four environments (S5 Table and Fig 3), which were distributed on 22 chromosomes of the linkage map of *G*. *hirsutum* × *G*. *barbadense* [12]. The mean phenotypic variation explained was 18.4%, with a range of 12 to 31.4%. Of the total, 15 marker loci were significantly associated with multiple QTL, which indicated that one gene may have multiple effects on different traits. In addition, 6 QTL were repeatedly detected in multiple environments, which were environment-stable QTL. These results provide valuable information for breeding.

Among the 84 significant marker-trait combinations, 15 marker loci associated with SI, in which 5 were repeatedly detected in 2 environments; 4 marker loci associated with BN; 7 marker loci associated with BW; 12 marker loci associated with LP; 6 marker loci associated with FE; 4 marker loci associated with MV; 8 marker loci associated with FS, in which NAU1148 on chromosome 11 was repeatedly detected in 2 environments; 4 marker loci associated with FUHML; 12 marker loci associated with FU; and 6 marker loci associated with PH.

A total of 58 markers were significantly associated with the traits, and 33 of them were EST-SSRs. After mapping these markers on the TM-1 genome sequence, 9 markers were found to be associated with genes located in the A and D sub-genome and contained 3 transcription factors (S6 Table).

### Discovery of elite alleles

In this study, elite allelic variation analysis of 84 significant trait-locus association combinations aimed to select excellent allelic variation from 3–79 and to apply these alleles in assisted selection of excellent germplasms. As shown in the S7 Table, the allelic loci associated with BW, SI, LP and FS were detected in 4 environments, including 15, 6, 3 and 7 allelic variations with positive effect, respectively, and 5, 1, 9 and 2 allelic variations with negative effect, respectively. The allelic loci associated with FE, MV, PH and FU were detected in 3 environments, including 2, 1, 1 and 7 allelic variations with positive effect, respectively, and 4, 3, 5 and 5 allelic variations with negative effect, respectively. The allelic loci associated with FUHML were detected in 2 environments, including one allelic variation with positive effect and 3 allelic variations with negative effect.

## Discussion

BILs have been important intermediate materials for genetic research and breeding practice. The populations of BC_1_, BILs and CSSLs constructed from *G*. *hirsutum* and *G*. *barbadense* have been reported [21–30]; the *G*. *hirsutum* varieties were TM-1 and SG747, and the *G*. *barbadense* varieties were Pima S-7 and Hai7124. Our research used Emian22, an upland cotton cultivar with high yield, moderate fiber quality and sensitive verticillium wilt; 3–79 is the genetic standard system of *G*. *barbadense*, with the characteristics of fine fiber quality and verticillium wilt resistance. They have been used as parents to build a BC_1_ mapping population to construct a high-density genetic linkage map [12]. Therefore, the results of this study provide strong support for research on marker-assisted selection to build CSSLs covering the whole genome, based on the two varieties. This study also presents a major resource for improving upland cotton by developing new germplasms.

### Phenotypic and genotypic diversity

Phenotypic diversity evaluation can be used to select elite germplasm with comprehensive performance and individual traits; and the genetic diversity of genotypes is reflected in the germplasm diversity. Thus, it is necessary to integrate them to improve the efficiency of molecular marker-assisted breeding and to fully understand the potential value of BILs in breeding and genetics research.

The breeding BILs with significant phenotypic variation contributed to select materials with valuable variation. Some materials with special phenotypes provided sources of variability for breeding varieties with high yield and photosynthetic efficiency. More importantly, plant type, leaf lobe type and leaf size are some of the major characteristics of high photosynthetic efficiency breeding; boll stalk length, boll type and boll size are the basis for evaluation of boll setting capability and yield potential for cotton varieties. These 11 BILs with special phenotype, shown in S2 Table, were biased towards their donor parent 3–79. This is valuable material for improving the traits of *G*. *hirsutum* by using *G*. *barbadense* with high photosynthetic efficiency and potential yield.

According to phenotypic clustering analysis of Euclidean distance, the phenotypic diversity among the 54 BILs was abundant. In fact, the germplasms applied in breeding require comprehensive performance and allow for integrated breeding goals. Therefore, the results of phenotypic clustering were used to analyze the relationship between the two lines, so that elite germplasms could be selected according to the goal of combination breeding.

In terms of molecular diversity of these BILs, a large variation in genetic similarities among the 54 BILs was observed, and the overall molecular genetic similarity was biased towards the receptor parent. BILs are also a primary population because there were many introgression fragments in the 54 BILs and the average background recovery rate was 79.8%. However, polymorphic loci in the BILs accounted for 84.4% of overall polymorphic loci, with MAF≥0.05, which indicated that multi-markers uniformly distributed in the genome could be used in QTL mapping. The high genetic diversity (I = 0.417) between the 54 BILs presented the possibility of selecting excellent germplasms. Moreover, 54 BILs were developed from the 200 original BC_1_ plants because of unavailable seeds, late maturation, weak vigor, etc. However, these did not affect the even distribution of introgression fragments along chromosomes. The introgression fragments totally covered approximately 5.86 times the total genomic length. There were un-introgression gaps or hot introgression regions according to our reference map which indicated that the phenomenon of marker segregation distortion improved the heterozygous genotype proportion in the population. Thus, the numerous heterozygous loci and homozygous 3–79 loci resulted in the introgression fragments covering the most of the genome after the selfing of 8 generations in the BC_1_F_8_.

### QTL mapping

In our research, association between phenotypic traits in multi-environments and molecular marker genotypes revealed some stable QTL detected in multi-environments and pleiotropic makers, which were the same as those reported by others [22, 23]. Meanwhile, 10 traits were not significantly associated with QTL, because many fragments were from the donor parent 3–79, and allelic variation of positive or negative effects might accumulate or offset one another, and the phenotypic differences existing in different genotype groups were small, which could lead to the decrease of QTL detection power.

Among these QTL markers (labeled neighbor markers), which were located in the genetic map at an average distance of 10 cM and associated with the same traits in our research, some tended to be associated with the same trait in the same environment, such as NAU2962-NAU3002, NAU3036-MUSS118, BNL1043-HAU1683, NAU3203-NAU3943, MGHES16-NAU6525, and BNL1040-BNL4079. Moreover, there were 3 other groups of markers, Gh075-NAU2962-NAU3002, HAU0567-CIR362, and NAU6582-TMB2762, associated with the same trait in different environments. In particular, BNL1043 was associated with SI in E1 and E2, and HAU1683, an adjacent marker, was associated with SI only in E1. The same result was also observed in the MGHES16-NAU6525 group. These results indicated that main effect QTL or genes in these chromosome regions should exist, and their effects may expand to larger chromosome regions so that they could be detected in these regions [31], These chromosome regions and marker loci stably detected in multi-environments could be considered important candidate regions in further studies by developing F_2_ and F_2:3_ populations or NILs (Nearly isogenic lines) for fine mapping these QTL.

None of the QTL detected in our research have been reported in previous studies, which is likely due to different markers used in QTL mapping as well as different background materials and environments. Moreover, compared to previous studies, some markers were associated with different traits. However, 3 of the 9 total annotated QTL related genes and their family members were reported, and they played important roles during cotton fiber development [32–37]. Moreover, 3 of these 9 related genes were transcription factors, which have recently become hotspots in plant growth and development research. Expression levels of these annotated genes were also obtained from RNA-seq data reported by Zhang et al. [18], and some of them were valuable compared with our results (S4 Fig). For example, NAU3203 was an EST-SSR associated with the PH in E2, while Gh_A13G2112 and Gh_D13G0451, two annotated genes of NAU3203 in the A and D sub-genome, respectively, were expressed abundantly in the stem of TM-1. It is possible that these identified QTL related genes alone are involved in the formation of cotton yield and fiber quality traits; alternatively, linkage or disequilibrium with upstream and downstream genes in the same chromosome region led to association with the target traits. In future research, to exclude false association and target genes, we could increase marker density or sequence large chromosome fragments surrounding the QTL region (approximately 100 kb) [38].

### Germplasm selection based on phenotypic and genotypic diversity in BILs

Eight potential breeding materials (Table 1) were selected according to phenotypic diversity, phenotypic clustering analysis, principal component analysis and positive alleles from 3–79. These materials not only had a single outstanding trait but also exhibited comprehensive performance. In these elite lines, there were two morphologically special materials, BS2 and BS49, indicating that agronomic traits from *G*. *barbadense* had been introgressed; meanwhile, they provided a valuable resource for plant type, high-efficiency photosynthesis breeding in cotton.

**Table 1:** Special BILs with outstanding traits and more positive alleles.

Line	Reference for selection	Outstanding traits		# Introgression loci	Markers associated with trait
BS2	Maximum FU in E1 and E2 with 3 FU positive alleles derived from 3–79.	FU (%)	86.7 (E1), 88.3 (E2), 85.3 (E3), 85.9 (E4)	27	HAU1683, MUSS281, NAU1148, NAU2602, NAU3036, NAU3481
BS3	Maximum FU in E4 and the fourth in E3 with 7 FU positive alleles derived from 3–79.	FU (%)	85.9 (E2), 86.0 (E3), 86.8 (E4)	39	BNL1043, BNL2443, BNL2895, BNL4079, DPL238, Gh185, HAU504, HAU1683, HAU2992, NAU1148, NAU2962, NAU3002, NAU3036, NAU3203, NAU3349, NAU3481, NAU5323, NAU5345, NAU6582
BS12	Maximum FE in 3 environments.	FE (%)	6.7 (E1), 5.6 (E2), 11.1 (E3)	26	BNL1434, BNL2443, BNL2895, DPL850, Gh199, Gh283, HAU1567, HAU3241, NAU1148, NAU2858, NAU6441
BS36	Maximum FUHML in E2, the second in E4, the third in E1 and E3.	FUHML (mm)	31.1 (E1), 32.0 (E2), 32.6 (E3), 31.0 (E4)	23	BNL663, BNL3436, Gh185, HAU504, HAU3132, NAU1148, NAU2858, NAU2962, NAU3002, NAU3349, NAU3684, NAU6525, MGHES16
BS41	Maximum FS in E2, the second in E1, the fifth in E4.	FS (cN/tex)	32.4 (E1), 34.9 (E2), 31.9 (E3), 29.3 (E4)	36	BNL252, CIR362, Gh697, HAU295, HAU567, HAU6992, MUSB0907, NAU1148, NAU2873, NAU2962, NAU3002, NAU3156, NAU3317, NAU3481, NAU5345, NAU6598
BS47	Maximum PH in 3 environments.	PH (cm)	92.0 (E1), 146.2 (E2), 83.0 (E4)	44	BNL252, BNL1379, BNL2443, BNL2895, BNL3436, CIR362, HAU295, HAU3132, MUSS563, MGHES16, NAU2602, NAU3481, TMB2901
BS49	Maximum SI in E1 and E2 with 11 SI positive alleles derived from 3–79.	SI (g)	13.3 (E1), 14.6 (E2), 10.8 (E3)	40	BNL1043, BNL1379, BNL2443, CIR362, DPL850, Gh183, NAU2858, HAU295, HAU1683, HAU3132, HAU3241, MGHES16, MUSS563, NAU2602, NAU2873, NAU3349, NAU5345, NAU6525, TMB2901
BS51	Maximum BN in three environments and maximum lint percentages in E1 and E4.	BN	44.1 (E1), 38.6 (E2), 6.8 (E4)	15	BNL3436, NAU2858

Through analysis of the genetic background of the eight excellent germplasms, we found that all of these lines introgressed more marker loci, including QTL associated with yield and fiber quality. BS3 and BS49 exhibited all the positive alleles related to FU and SI, respectively, and their corresponding traits were outstanding (Table 1). The two lines not only had the breeding potential for high fiber quality and yield but also could be directly applied in breeding as excellent germplasm.

In conclusions the BILs developed in this study had various phenotypic and molecular variations and should be considered valuable resources for cotton genetics and breeding.

## Supporting Information

S1 Fig Phenotype of fuzz color of some BILs. (TIF)

S2 Fig Clustering tree of phenotypic Euclidean distance. (TIF)

S3 Fig UPGMA clustering based on genetic similarity coefficients of molecular markers. (TIF)

S4 Fig FPKM of annotated genes derived from reported RNA-seq data [17]. FPKM: expected number of fragments per kilobase of transcript sequence per millions of base pairs sequenced.
(TIF)

S1 Table Statistical summary of different traits of the 54 BILs in 4 environments. (XLS)

S2 Table Morphological and plant architecture traits of the special BILs. (XLS)

S3 Table Length and number of introgression fragments on chromosomes. (XLS)

S4 Table The analysis of segregation distortion of polymorphic markers in BILs. (XLS)

S5 Table Marker-trait associations derived from the null hypothesis for multiple testing with a FDR-corrected threshold at the P≤0.01 level. (XLS)

S6 Table The results of comparative mapping makers’ sequence to TM-1 genome sequence. (XLS)

S7 Table Phenotypic effect value of alleles derived from 3–79. (XLS)
