# Supplementary figures and images for: A BIL Population Derived from G. hirsutum and G. barbadense Provides a Resource for Cotton Genetics and Breeding
Source: PLoS One. 2015 Oct 30;10(10):e0141064. doi: 10.1371/journal.pone.0141064 (PMC4627741; doi:10.1371/journal.pone.0141064)

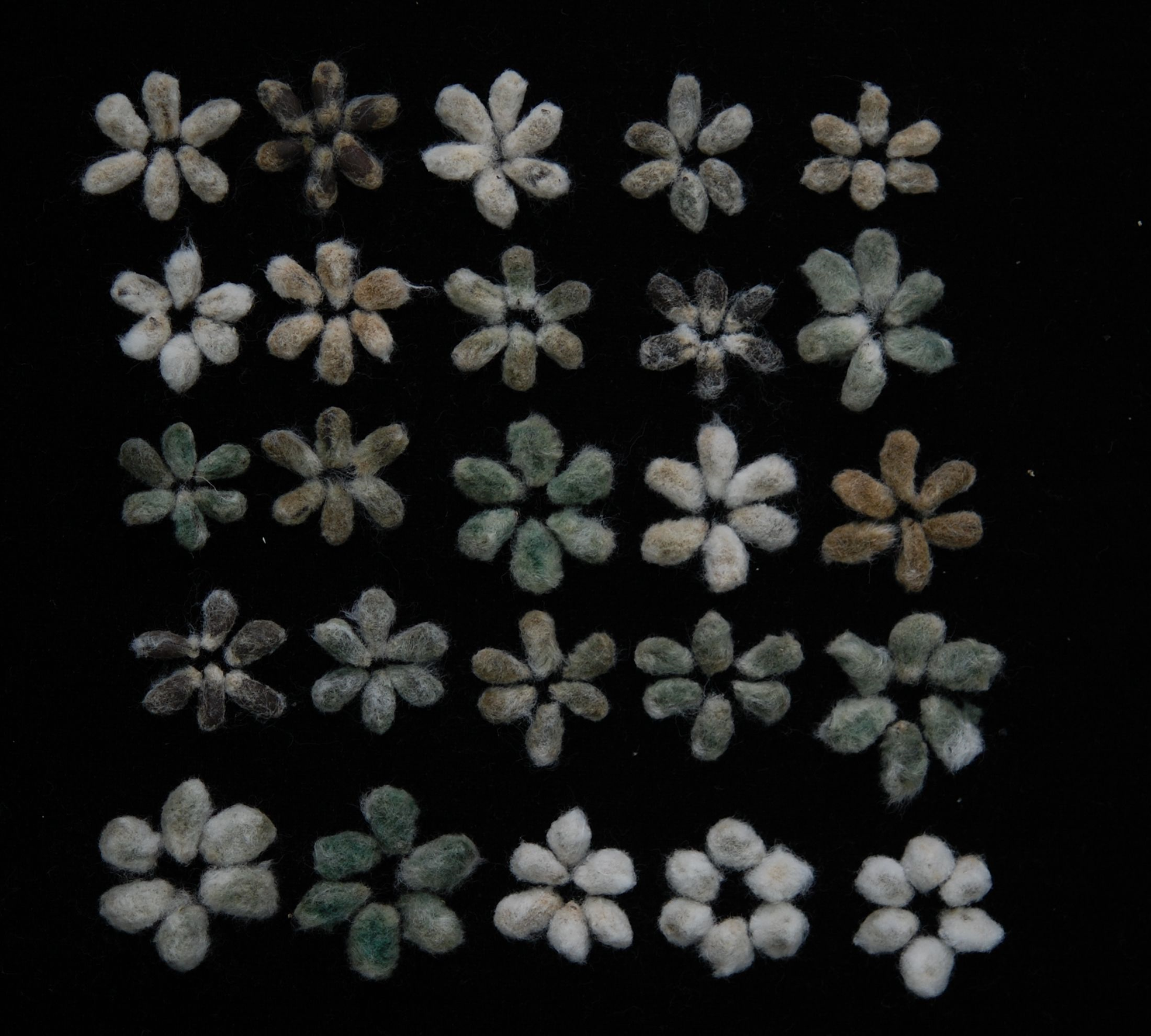

Supplement: S1 Fig — (TIF) [file pone.0141064.s001.tif]

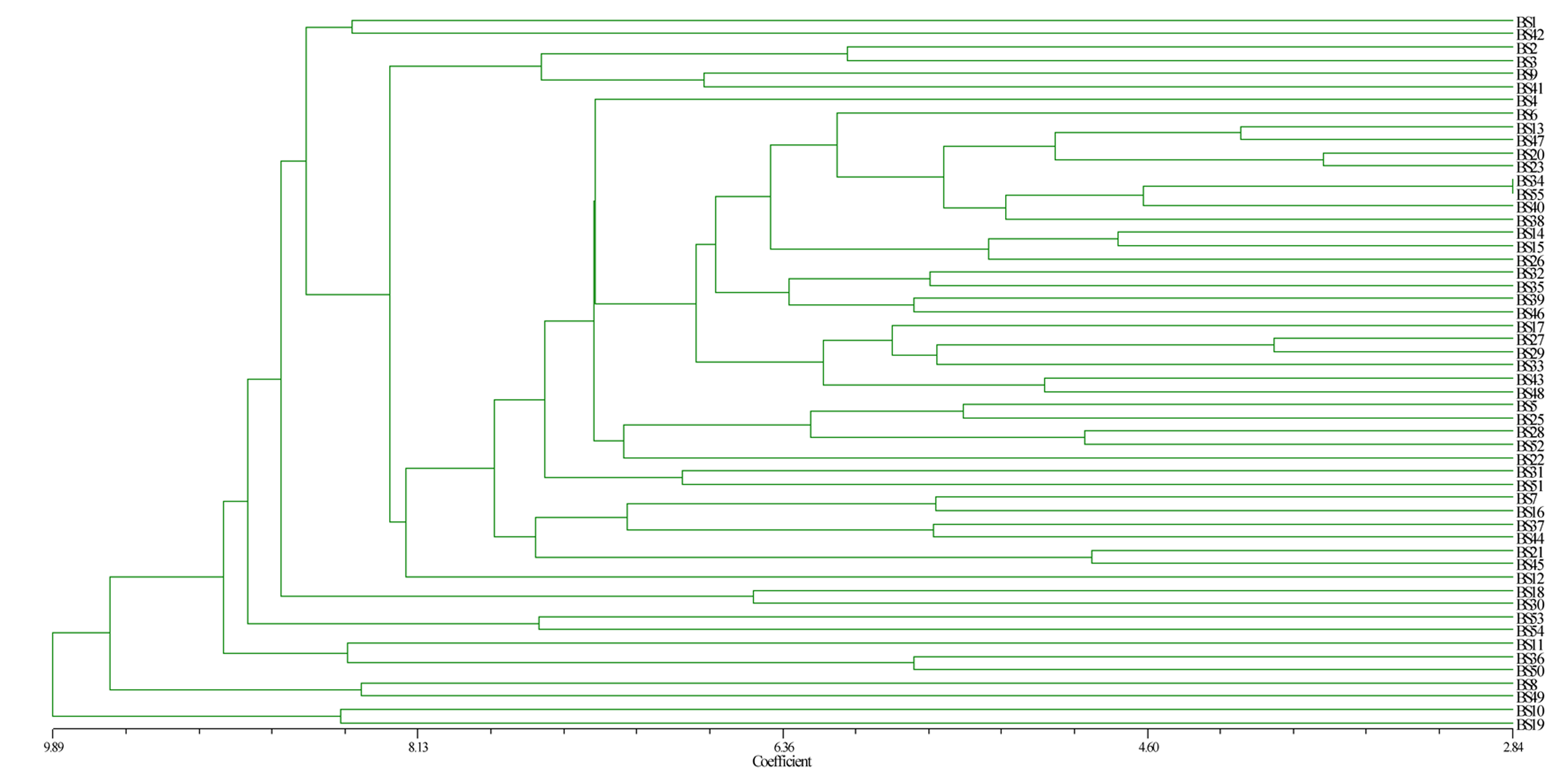

Supplement: S2 Fig — (TIF) [file pone.0141064.s002.tif]

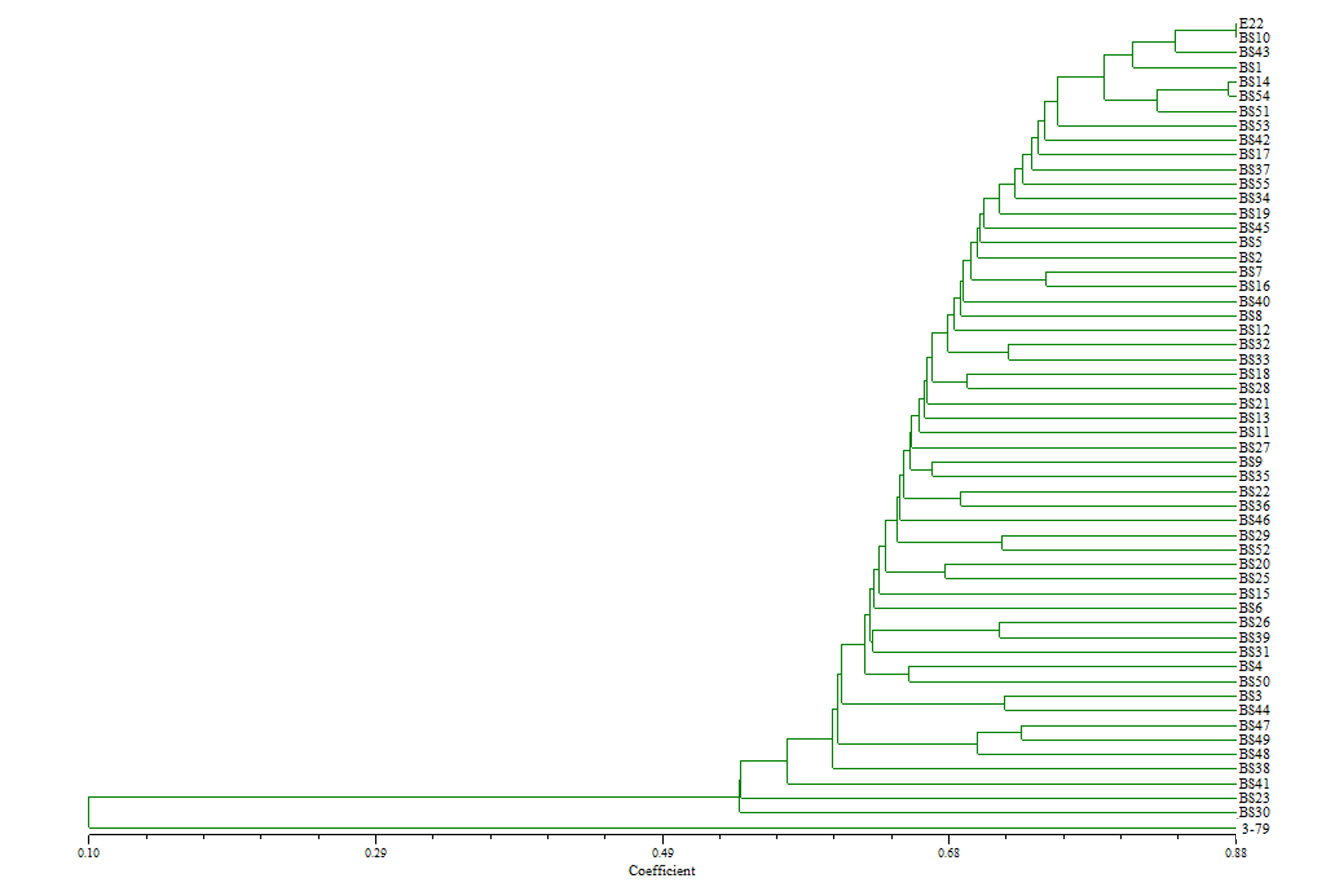

Supplement: S3 Fig — (TIF) [file pone.0141064.s003.tif]

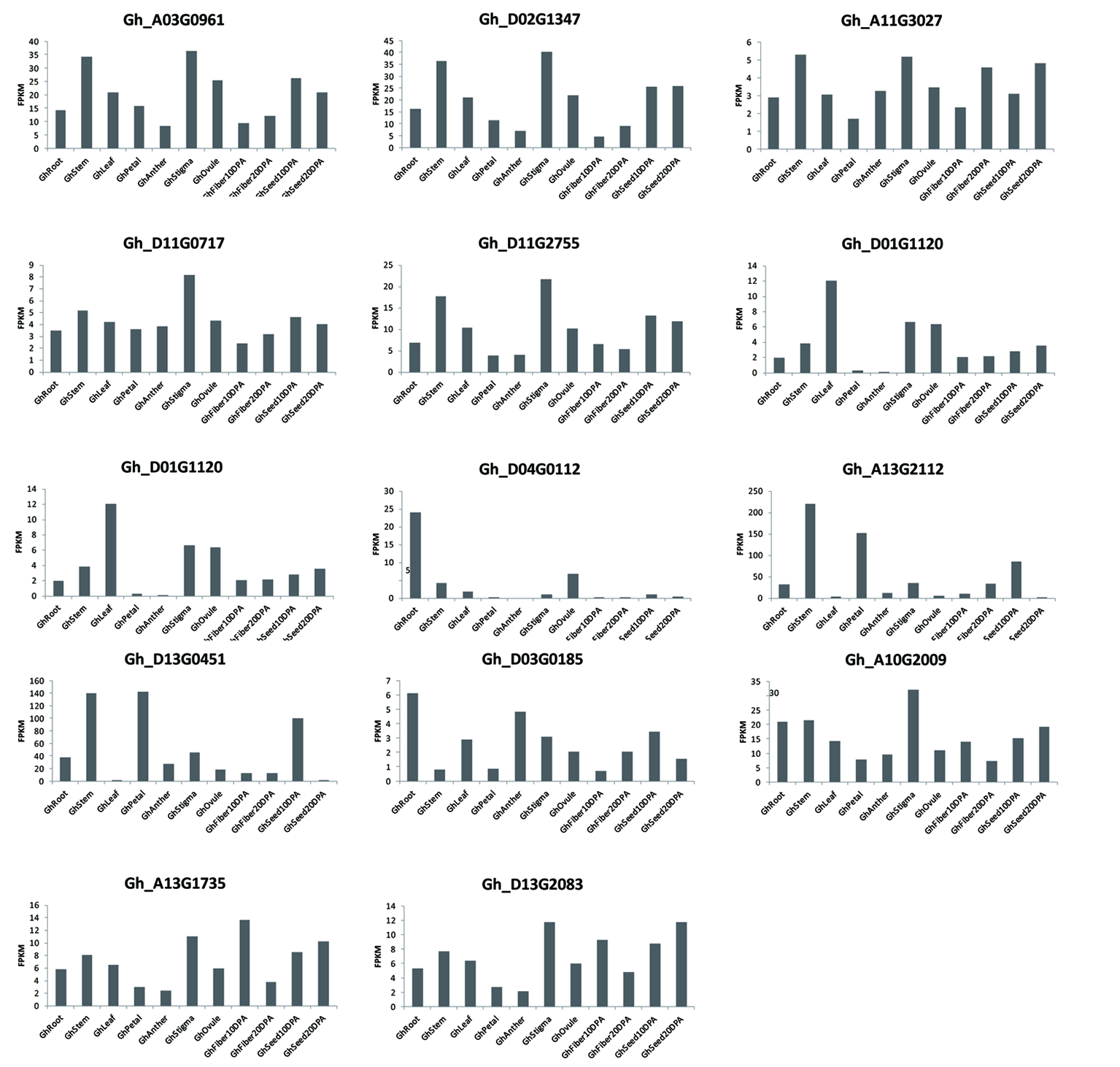

Supplement: S4 Fig — FPKM: expected number of fragments per kilobase of transcript sequence per millions of base pairs sequenced. (TIF) [file pone.0141064.s004.tif]
